# Renal Arteriography via Radial Artery Access with a 125 cm Long Angiographic Catheter

**DOI:** 10.1155/2021/5564462

**Published:** 2021-04-23

**Authors:** Ji-Xuan Liu, Zhi-Jun Sun, Jin-Da Wang

**Affiliations:** ^1^Department of Cardiology, Beijing Friendship Hospital, Capital Medical University, Beijing 100050, China; ^2^Department of Cardiology, The Sixth Medical Centre of PLA General Hospital, Beijing 100853, China

## Abstract

A 125 cm long catheter makes it possible to perform renal arteriography via radial artery, but its feasibility and safety remain unclear. Our study recruited 1,323 patients grouped by two different vascular accesses to renal arteriography, i.e., femoral artery access and radial artery access. The success rate of angiography was 100% in both groups. Differential analysis showed that the overall complication incidence of radial artery access group was significantly lower (2.5% for radial artery access vs. 4.8% for femoral artery access, *p* = 0.03). From this study, we suggest that using the 125 cm angiographic catheter to perform renal arteriography via radial artery access is feasible and safe.

## 1. Introduction

Atherosclerosis is the main cause of renal artery stenosis. Like other atherosclerotic diseases, the incidence of atherosclerotic renal artery stenosis (ARAS) rises with the progress of population aging [1, 2]. In fact, there exists a high rate of diagnostic omission errors for ARAS because asymptomatic patients usually cannot receive timely treatment until renal function deteriorates and cardiovascular injuries occur [3]. Therefore, early diagnosis becomes significantly important to the prognosis of patients with ARAS.

Renal arteriography (RAG) is the gold standard for diagnosing renal artery stenosis [4, 5]. The femoral artery is the traditional access of renal arteriography and is also preferred by most hospitals and interventional doctors. However, the femoral artery access (FAA) can cause strong discomfort, high incidence of complications such as hematoma and bleeding, and has been almost completely replaced by radial artery access (RAA) in the coronary intervention [6]. Moreover, 11.3 ~ 39% of coronary artery disease patients are complicated with ARAS [7]. It was reported that the 125 cm long angiographic catheter can make it easily accessible for the operation [8]. Therefore, this study is aimed at investigating the feasibility and safety of RAG via the RAA. The results would increase the diagnostic rate of ARAS.

## 2. Materials and Methods

### 2.1. Study Design

Our study was designed according to the reported standard of the observational study. We searched 1,323 consecutive patients admitted to the Department of Cardiology at the Chinese PLA General Hospital and extracted RAG from January 2016 to December 2020 in the PACS database. All subjects were grouped by different vascular access (decided by the experienced interventional cardiologists according to their own operation habit). The index related to RAG operation and the incidence of complications between the two groups were compared. This study was approved by the Ethics Committee of Chinese PLA General Hospital. All participants were called for verbal agreements.

### 2.2. Angiography Procedures

In the FAA group, either right or left femoral artery puncture was first performed; then, a 6F sheath was inserted. 2500 units of unfractionated heparin were injected into the artery to prevent catheter-related thrombosis. A 6F Judkins R angiography catheter was inserted through the femoral artery sheath to the level of the first lumbar vertebra, and renal arteriography was implemented at the opening of the renal artery.

In the RAA group, after puncture was performed at the right or left radial artery, the 6F arterial sheath was inserted. 2500 units of heparin were injected into the artery to prevent catheter-related thrombosis and followed by 200 *μ*g nitroglycerin to prevent vasospasm. Guided by the guidewire, a 6F and 125 cm long MP A1 angiography catheter (Cordis, USA) was put into the ascending aorta. The open end of the catheter was turned to the left of the patient at this time. At last, the guidewire was sent into the descending aorta, through which the catheter can be sent into the opening of the renal artery.

### 2.3. Data Collection

All demographic information was collected from the electronic medical record system in a standard report form, such as age, gender, height, and RAG-related data including X-ray irradiation time, contrast agent dosage, and complication incidence.

### 2.4. Data Analysis

SPSS version 20.0 was used to analyze all the data of our study. All statistics were two-sided tested at the 5% level of significance. All results were reported referring to STROBE statement. Continuous data with normal distribution and homogeneity of variance were compared by one-way ANOVA, otherwise by Mann–Whitney test. Categorical data were compared by *χ*^2^ analysis.

## 3. Results

### 3.1. Baseline Characteristics

From 1,323 patients enrolled in our study, renal arteriography was performed in 612 patients (mean age of 61.3 ± 17.7, 62.9% male, mean height of 166.3 ± 9.9 cm, and mean serum creatinine 87.6 ± 21.4 ummol/L) via RAA and 711 patients (mean age of 62.1 ± 18.1, 59.3% male, mean height of 168.5 ± 9.7 cm, and mean serum creatinine 89.2 ± 22.3 ummol/L) via FAA. There presented no significant difference between the two groups in baseline characteristics (Table 1).

### 3.2. RAG Operation Related Index

In the RAA group, all 612 patients underwent angiography successfully via radial artery. The right radial artery was initially selected in 464 patients and the left radial artery for the others (*N* = 148). Among the patients who initially chose the right radial artery, 14 patients were taken another puncture of the left radial artery because the aortic arch was too tortuous so that the catheter could not be adjusted to the descending aorta to complete the RAG. In the FAA group, all 711 patients underwent angiography through femoral artery access without changing the punctured vessel. If the frequency of puncture is not considered, there is no difference of the success rate of angiography between the two vascular accesses (both 100%).

The differential analysis shows that operation time (5.08 ± 1.75 min for FAA vs. 8.96 ± 2.03 min for RAA, *p* = 0.01) and X-ray exposure time (2.11 ± 0.16 min for FAA vs. 4.13 ± 0.23 min for RAA, *p* = 0.03) of FAA were significantly shorter than that of the RAA group. However, there was no significant difference in the dosage of contrast (15.19 ± 3.38 mL for RAA vs. 14.38 ± 3.69 mL for FAA, *p* = 0.31) and the change of serum creatinine (9.32 ± 7.94 ummol/L for RAA vs. 9.38 ± 8.09 ummol/L for FAA, *p* = 0.82) between the two groups (Table 2).

### 3.3. Complications

*χ* ^2^ analysis showed that the incidence of complications in the RAA group was not higher than that in the FAA group. Although there was no significant difference in single complication (including aortic artery dissection, renal artery dissection, retroperitoneal hematoma, hematoma, pseudoaneurysm, and arteriovenous fistula), the overall complication incidence of the radial artery group was significantly lower (2.5% for RAA vs. 4.8% for FAA, *p* = 0.03) (Table 3).

## 4. Discussion

In this retrospective cohort study, we found that the success rate of renal arteriography with the 125 cm long catheter via RAA was 100%. Despite slightly more operation and line exposure time, the complication rate of renal arteriography via radial artery was 48% less than that via the femoral artery.

Although previous studies have confirmed the possibility of renal arteriography via radial artery [8–10], few have compared the feasibility and safety between RAA and FAA [11]. Recently, a comparative study for noncoronary angiography with a small number of renal angiography cases has reported that radial artery is the feasible and safe access [12], which is consistent with our results.

The puncture point of the femoral artery approach is short from the opening of the renal artery, and the access is relatively straight. This anatomical feature makes the transfemoral artery approach easy to operate and keep the standard way of RAG. In our study, results related to RAG operation such as operation time and X-ray exposure time have reflected this feature. However, femoral artery access has inherent disadvantages as increased bleeding-related complications and patient discomfort [4, 5, 13]. The 125 cm long angiographic catheter enables enough distance for the RAG via RAA or even the right RAA. This study revealed that the success rate of renal arteriography via the right radial artery was up to 97% (464 in total, 450 patients succeeded, and 14 patients failed due to extreme distortion of the aortic arch).

It should be noted that in our study, a male with a height of 195 cm successfully received RAG via the right radial artery, but the 125 cm long catheter was completely inserted into the sheath of the radial artery. This case suggests that patients taller than a certain threshold should not receive RAG with the 125 cm long catheter, which should be further studied.

The limitations of our study included the recall bias and single-center study. More analysis should be conducted on data related to renal artery intervention and simultaneous coronary and renal angiography.

## 5. Conclusion

This study has shown that it is feasible and safe to use the 125 cm long angiographic catheter for renal arteriography via radial artery access. Radial artery vascular access can be the first choice for RAG in clinics.

## Figures and Tables

**Table 1:** Baseline characteristics of patients grouped by different vascular access.

Characteristics^∗^	RAA (*N* = 612)	FAA (*N* = 711)	*p* ^†^
Age, years	61.3 ± 17.7	62.1 ± 18.1	0.66
Male, no. (%)	385 (62.9)	422 (59.3)	0.18
Height, cm	166.3 ± 9.9	168.5 ± 9.7	0.42
Scr, ummol/L	87.6 ± 21.4	89.2 ± 22.3	0.42

^∗^Categorical variables are shown as numbers (%) and continuous variables as means ± standard deviation. ^†^*p* values were calculated by comparing characteristics between two groups. RAA: radial artery access; FAA: femoral artery access; Scr: serum creatinine.

**Table 2:** Comparison of RAG-related index between different vascular access.

Item^∗^	RAA (*N* = 612)	FAA (*N* = 711)	*p* ^†^
Success rate, %	100	100	1
Operation time, min	8.96 ± 2.03	5.08 ± 1.75	0.01
X-ray exposure time, min	4.13 ± 0.23	2.11 ± 0.16	0.03
Contrast, mL	15.19 ± 3.38	14.38 ± 3.69	0.31
Change of Scr, ummol/L	9.32 ± 7.94	9.38 ± 8.09	0.82

^∗^Categorical variables are shown as numbers (%) and continuous variables as means ± standard deviation. ^†^*p* values were calculated by comparing characteristics between two groups. RAG: renal arteriography; RAA: radial artery access; FAA: femoral artery access; Scr: serum creatinine.

**Table 3:** Comparison of complications between different vascular access.

Complications^∗^	RAA (*N* = 612)	FAA (*N* = 711)	*p* ^†^
Aortic artery dissection, no. (%)	0 (0)	0 (0)	1
Renal artery dissection, no. (%)	0 (0)	0 (0)	1
Retroperitoneal hematoma, no. (%)	0 (0)	0 (0)	1
Hematoma, no. (%)	11 (1.8)	25 (3.5)	0.06
Pseudoaneurysm, no. (%)	3 (0.5)	6 (0.8)	0.44
Arteriovenous fistula, no. (%)	1 (0.2)	3 (0.4)	0.39
Total, no. (%)	15 (2.5)	34 (4.8)	0.03

^∗^Complications are shown as numbers (%). ^†^*p* values were calculated by comparing characteristics between two groups. RAG: renal arteriography; RAA: radial artery access; FAA: femoral artery access.

## Data Availability

The cohort study data used to support the findings of this study are restricted by the Ethics Committee of Chinese PLA General Hospital in order to protect patient privacy.
